# In Which Scenarios Do Preceptors and Students Agree, Disagree, or Remain Neutral About Learner Mistreatment?

**DOI:** 10.1007/s40670-024-02261-z

**Published:** 2025-01-09

**Authors:** Alejandra Colón-López, Anne Zinski

**Affiliations:** Department of Medical Education, University of Alabama at Birmingham Heersink School of Medicine, 600W9 VH 16670 University Blvd, Birmingham, AL 35233 USA

**Keywords:** Learner mistreatment, Vignettes, Medical students, Preceptors

## Abstract

**Objective:**

In recent years, nearly half of graduating medical students in the USA and Canada reported personal mistreatment experiences during training. Prior scholarship reports heterogeneous opinions of learner mistreatment behaviors among trainees, and resulting unaligned perceptions may influence reporting, feedback, and policy. However, fewer studies compare students’ and preceptors’ views about learner mistreatment using vignettes of student-preceptor interactions.

**Methods:**

We surveyed 141 students and 203 preceptors at an MD-granting institution. Participants indicated their agreement on a 5-point scale on whether behaviors in 17 written vignettes constituted learner mistreatment. Descriptive statistics and bivariate tests were executed to identify areas in which students’ and preceptors’ mistreatment views differed.

**Results:**

Student and preceptor responses converged on 12 of 17 vignettes. More students agreed that vignettes describing a student being addressed by another student’s name and an example of public embarrassment constitutes learner mistreatment. More preceptors agreed that addressing students with terms of endearment and denying training opportunities based on career choice constitute learner mistreatment. The proportion of preceptors who selected neutral responses was higher than that of students for nearly all vignettes.

**Conclusion:**

When presented with written vignettes, students’ and preceptors’ perceptions of learner mistreatment were not consistent. This study highlights potential gaps in students’ and preceptors’ mistreatment perceptions, including differences in participants’ decisions to remain “neutral.” To prevent and address learner mistreatment, follow-up research is warranted to support the development and honing of shared definitions and examples of mistreatment, as well as targeted programming for students and preceptors.

## Introduction

The continued prevalence of learner mistreatment in medical education remains a pressing issue in learning environment research and institutional reports [1–6]. In each year since 2018, approximately 40% of graduating medical students in the USA and Canada reported personal experiences with at least one of 16 negative behaviors, excluding public embarrassment, as defined by the Association of American Medical Colleges (AAMC), and 88% of these reported behaviors occurred in clinical settings [7]. Mistreatment has been shown to have direct detrimental effects on the learning environment as a whole, affecting medical students’ content learning, skill acquisition, and confidence [8, 9]. Additional evidence has linked reconsideration of specialty choice and reports of poor mental health to learner mistreatment [10–13]. Furthermore, physicians who report a history of experiencing mistreatment as trainees may be more likely to mistreat patients and healthcare team members [14].

Previous research shows that learners have different opinions of what constitutes mistreatment, and this lack of consensus likely influences reporting decisions [15–18]. Beyond fear of retaliation and lack of trust in processes for addressing problems in the learning environment, students’ reporting decisions are also susceptible to their peers’ and preceptors’ ability to recognize mistreatment behaviors [19, 20]. While medical schools often employ robust reporting systems to monitor the learning environment and act upon reported mistreatment behaviors [20], differing views of whether an event constitutes learner mistreatment are likely to affect awareness, reporting, and potential for intervention [18, 20, 21]. According to Bell et al. [19], peer and faculty feedback can also shape students’ interpretation of acceptable behaviors in the learning environment, and unaligned views can discourage students from reporting mistreatment incidents [22].

To date, very few studies examine whether learner views of mistreatment differ from those of their faculty preceptors [16, 23]. However, Peckston et al. [24] demonstrated differences in faculty and student perceptions of mistreatment related to gender, career choice, public humiliation, and requests to perform unreasonable tasks, providing compelling rationale to further explore these differences. In this study, we use vignettes that illustrate an array of interactions between students and faculty, residents, or other healthcare professionals to examine whether students and preceptors perceive mistreatment differently. Additionally, we consider differences in participants’ neutral responses to vignette cases, as they may reflect uncertainty about mistreatment and/or willingness to reconsider their initial viewpoints [15, 23, 25–27].

Vignettes are widely used for evaluating adult perceptions of behavior as well as instructional tools for cultivating awareness among faculty and trainees, and prior work shows the value of utilizing written cases and descriptive vignettes to evaluate individual views of mistreatment [15–17, 22–27]. This study utilizes 17 written vignettes to examine the perceptions among students and preceptors to address two aims: (1) assess medical students and preceptors’ perceptions of mistreatment, including neutral views, and (2) determine which behaviors yield differing views between these two groups.

## Materials and Methods

### Survey Setting

In February 2023, we invited medical students, graduate trainees, and faculty from an academic medical center in the Southeastern USA with three regional clinical campus sites to complete an online cross-sectional survey about the learning environment. Participants had the opportunity to submit their responses within a 5-week window. Recruitment consisted of invitations from the school’s leadership team, emailed invitations, and newsletter advertising sent to undergraduate medical students, faculty, and graduate trainees. Eligible participants received three reminder emails while the survey was open. Respondents completed an informed consent process prior to completing the survey. The Institutional Review Board at the investigators’ home institution provided ethical review for this study.

### Data Collection Instrument

Written vignettes were designed to evaluate and compare preceptors’ and students’ perceptions of learner mistreatment. The vignettes in this study reflected commonly reported mistreatment behaviors between 2020 and 2022 at the selected institution [28], as well as areas of concern that were reported via a series of discussions with local and regional campus deans, administrators, student services representatives, and students. The authors also reviewed previously published written and video vignettes, as well as training resources used in prior studies, eventually selecting and/or adapting nine previously tested and published cases [15, 16, 23, 24, 29, 30]. Based on qualitative excerpts from Kristoffersson and colleagues [31], as well as input from our institution’s recent graduates, ten additional vignettes were generated.

The authors agreed to explore two main areas of mistreatment behaviors. The first list included behaviors that were reported by recent graduating students on the local AAMC GQ survey: denied training or reward opportunities based on gender; public embarrassment or humiliation; and negative or offensive behaviors based on characteristics other than gender, race/ethnicity, or sexual orientation. The second list included behaviors used in prior publications or those reported locally that were not included in the AAMC list of negative behaviors [32]. This combination contributed to a preliminary list of potential vignettes for this study.

Next, authors iteratively refined each case description for piloting. Two students and one faculty educator provided extensive feedback on 19 pilot vignettes, including one “control” vignette. In subsequent discussions, two cases were removed from the preliminary list, and the sequence in which they appeared in the survey was arranged based on length and portrayed behavior. The final list included 17 vignettes; six cases described behaviors that reflected AAMC-defined “negative behaviors” criteria [32] and 11 cases did not strictly meet these definitions.

The authors used the Qualtrics Survey application to construct and disseminate the survey tool [33]. Participants first indicated their role (i.e., medical student, faculty, resident or fellow, other), and whether they had received training related to the learning environment. For the subsequent portion of the survey, participants were asked to indicate on a 5-point Likert scale the extent to which they agreed or disagreed that each written vignette demonstrated learner mistreatment. In the closing questions, participants reported their gender identity and year of medical school training (MS) or years of teaching experience.

### Statistical Analysis

For each vignette, we assigned values of 1–5 to each Likert-type scale category: 1 for “Strongly disagree,” 2 for “Disagree,” 3 for “Neutral,” 4 for “Agree,” and 5 “Strongly agree” [16, 22–24, 27]. Categories in each vignette were subsequently recoded to generate a three-category outcome for each vignette 1 for “Strongly disagree or Disagree,” 1 for “Neutral,” and 3 for “Agree or Strongly agree” [24, 34]. To examine “Neutral” responses specifically, an additional binary variable was generated: 1 for “Strongly disagree, Disagree, Agree, or Strongly agree” and 2 for “Neutral.”

Due to low participation of respondents who identified their role as “Resident or Fellow” and “Other,” we combined these observations with “Faculty” observations, as they would represent the preceptor role. This resulted in two groups of respondents classified as 1 for “Student” and 2 “Preceptor.”

Descriptive statistics were generated to summarize demographic characteristics and responses to vignettes for students and preceptors separately. Listwise deletion was applied to address challenges associated with missing data. This approach yielded an analytic sample of 271 complete observations.

Fisher’s exact tests with Pagano and Halvorsen-computed *p* values [35] were employed to test differences in the proportions of students’ and preceptors’ Likert-type scale responses for each vignette. Additional Fisher’s exact tests were executed for vignettes that yielded significant results in initial bivariate testing to determine the difference in proportions of “strongly agree or agree” responses between students and preceptors. An additional set of Fisher’s exact tests was performed to test the difference in proportions of students and preceptors who selected “neutral” in each vignette [36]. All statistical analyses were performed using Stata/SE 18 [37].

## Results

A total of 351 participants (141 medical students; 203 preceptors) responded to the survey. Table 1 presents the demographic characteristics of medical students (*n* = 100) and preceptors (*n* = 171) who completed the entire survey. At the time of the survey, 89% of student respondents identified as male (42%) or female (47%), and 92% of preceptors identified as male (56%) or female (36%). More than half of students identified as first or second year students (MS1 29%; MS2 28%), and 38% identified as a third or fourth year medical students. One-third of preceptors reported teaching only in the clinical setting (35%), another third reported teaching in both pre-clinical and clinical settings (33%), 14% provided pre-clinical training only, and 9% were not teaching medical students at the time that they completed the survey.

**Table 1:** Sample characteristics (*N* = 271)

Student (*N* = 100)		Preceptor (*N* = 171)	
	(%)		(%)
Gender		Gender	
Male	42 (42)	Male	96 (56)
Female	47 (47)	Female	61 (36)
Non-binary or non-conforming	3 (3)	Non-binary or Non-conforming	1 (1)
Other	0 (0)	Other	1 (1)
Prefer not to say	8 (8)	Prefer not to say	12 (7)
Medical school year		Teaching setting	
MS1	29 (29)	Pre-clinical only	24 (14)
MS2	28 (28)	Pre-clinical and clinical	57 (33)
MS3	24 (24)	Clinical only	60 (35)
MS4	14 (14)	I don't teach students	16 (9)
Graduate program or other leave	5 (5)	Prefer not to say	14 (8)

*N*, observations; *MS1*, first year; *MS2*, second year; *MS3*, third year; *MS4*, fourth year

Table 2 presents the proportions of medical students’ and preceptors’ responses to each written vignette. Over 75% of both preceptors and students agreed that five of the 17 vignettes (V5, V10, V11, V15, and V16) constituted learner mistreatment. Most preceptors also agreed that behaviors described on V2 and V9 constituted learner mistreatment. Nearly all students agreed that the behaviors in V4 constituted learner mistreatment. Preceptors (86%) and students (82%) also responded similarly to V11 (control vignette).

**Table 2:** The proportion of students’ and preceptors’ (i.e., Faculty, Residents, Fellows, and Other) Likert-scale responses to each vignette and results from Fisher’s exact tests determining difference in students’ and preceptors’ perceptions in each vignette (*n* = 271)

Vignette	Student	Preceptor	*p* value
	(*N* = 100)	(*N* = 171)	
**V1: Limited Ob/Gyn participation for male student (%)**			0.092
** Strongly disagree or** disagree	37 (37)	42 (25)	
** Neutral**	19 (19)	36 (21)	
** Agree or** strongly **agree**	44 (44)	93 (54)	
**V2: "Don't worry, Honey." (%)**			**0.001**
** Strongly disagree or** disagree	25 (25)	14 (8)	
** Neutral**	16 (16)	25 (15)	
** Agree or** strongly **agree**	59 (59)	132 (77)	
**V3: Limited OR participation for female student (%)**			0.789
** Strongly disagree or** disagree	13 (13)	27 (16)	
** Neutral**	15 (15)	27 (16)	
** Agree or** strongly **agree**	72 (72)	117 (68)	
**V4: "You should know the answer to that." (%)**			**0.035**
** Strongly disagree or** disagree	11 (11)	34 (20)	
** Neutral**	14 (14)	35 (21)	
** Agree or** strongly **agree**	75 (75)	102 (60)	
**V5: Student’s preferred pronouns (%)**			1.000
** Strongly disagree or** disagree	6 (6)	10 (6)	
** Neutral**	6 (6)	11 (6)	
** Agree or** strongly **agree**	88 (88)	150 (88)	
**V6: EKG interpretation (Control) (%)**			0.347
** Strongly disagree or** disagree	82 (82)	147 (86)	
** Neutral**	12 (12)	12 (7)	
** Agree or** strongly **agree**	6 (6)	12 (7)	
**V7: "I bet you were a great athlete." (%)**			0.602
** Strongly disagree or** disagree	18 (18)	24 (14)	
** Neutral**	23 (23)	46 (27)	
** Agree or** strongly **agree**	59 (59)	101 (59)	
**V8: Family planning and career choice (%)**			0.501
** Strongly disagree or** disagree	53 (53)	79 (46)	
** Neutral**	25 (25)	53 (31)	
** Agree or** strongly **agree**	22 (22)	39 (23)	
**V9: Persisting approach from faculty (%)**			** < 0.001**
** Strongly disagree or** disagree	16 (16)	3 (2)	
** Neutral**	15 (15)	9 (5)	
** Agree or** strongly **agree**	69 (69)	159 (93)	
**V10: Misleading report from resident (%)**			0.101
** Strongly disagree or** disagree	2 (2)	14 (8)	
** Neutral**	5 (5)	10 (6)	
** Agree or** strongly **agree**	93 (93)	147 (86)	
**V11: Caregiving tasks (%)**			0.425
** Strongly disagree or** disagree	8 (8)	7 (4)	
** Neutral**	16 (16)	28 (16)	
** Agree or** strongly **agree**	76 (76)	136 (80)	
**V12: Limited OR participation due to career choice (%)**			**0.022**
** Strongly disagree or** disagree	30 (30)	30 (18)	
** Neutral**	23 (23)	33 (19)	
** Agree or** strongly **agree**	47 (47)	108 (63)	
**V13: Orientation for selected group of students (%)**			0.237
** Strongly disagree or** disagree	42 (42)	78 (46)	
** Neutral**	26 (26)	54 (32)	
** Agree or** strongly **agree**	32 (32)	39 (23)	
**V14: Wrong student name (%)**			**0.017**
** Strongly disagree or** disagree	7 (7)	28 (16)	
** Neutral**	25 (25)	54 (32)	
** Agree or** strongly **agree**	68 (68)	89 (52)	
**V15: Manuscript authorship (%)**			0.059
** Strongly disagree or** disagree	3 (3)	2 (1)	
** Neutral**	4 (4)	1 (1)	
** Agree or** strongly **agree**	93 (93)	168 (98)	
**V16: Interjecting feedback-English fluency (%)**			0.067
** Strongly dis**agree or disagree	4 (4)	12 (8)	
** Neutral**	8 (8)	28 (16)	
** Agree or** strongly **agree**	88 (88)	131 (77)	
**V17: Missed training opportunity (%)**			0.210
** Strongly disagree or disagree**	37 (37)	47 (28)	
** Neutral**	20 (20)	46 (27)	
** Agree or strongly agree**	43 (43)	78 (46)	

*n*, observations; *Ob/Gyn*, obstetrics and gynecology; *EKG*, electrocardiogram; *OR*, operating room

**Fig. 1:**
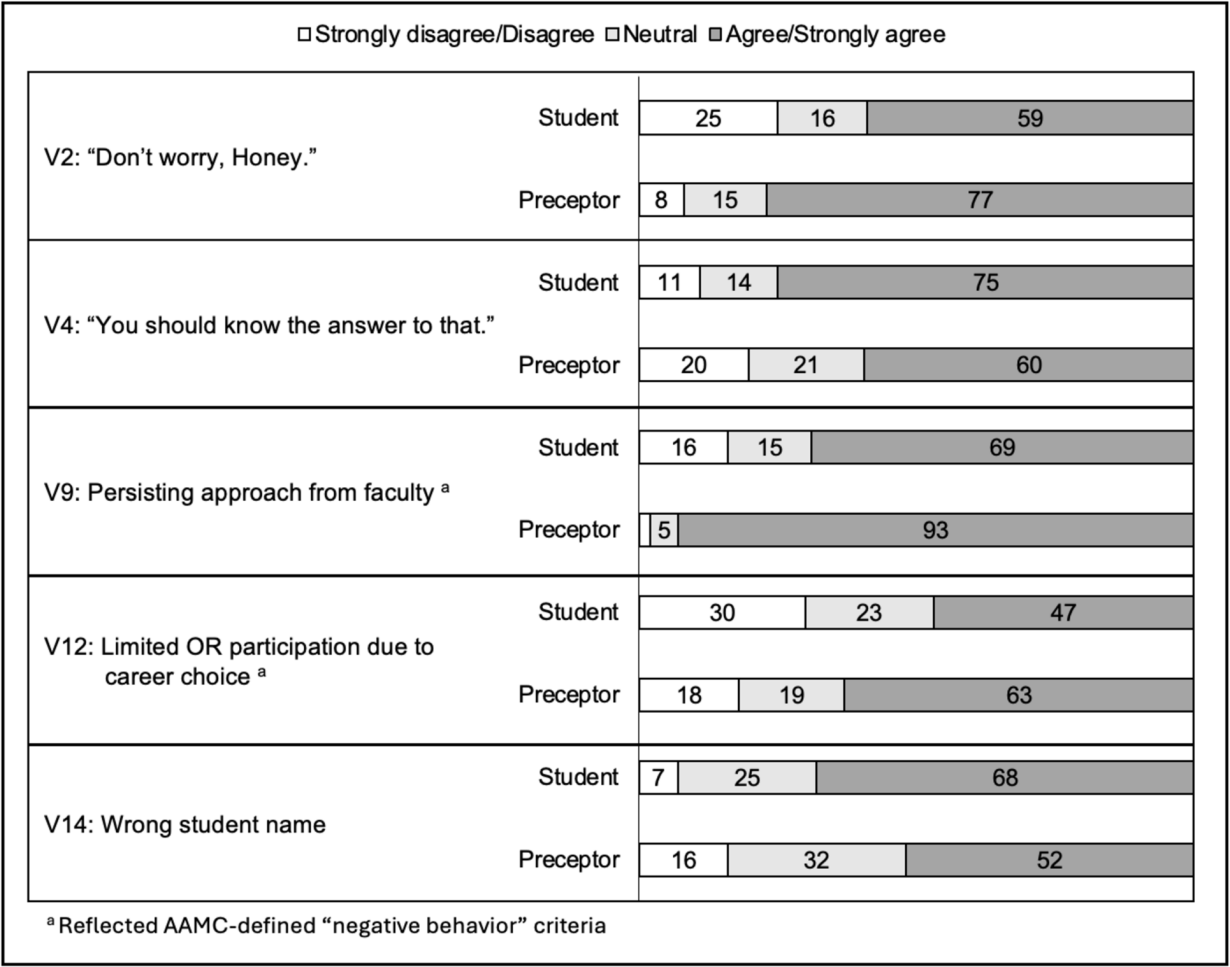
Student and preceptor perceptions on five vignettes with significantly different responses

Student and preceptor perceptions differed significantly on five of the 17 vignettes: V2 (*p* = 0.001), V4 (*p* = 0.035), V9 (*p* < 0.001), V12 (*p* = 0.022), and V14 (*p* = 0.017) (see Table 2 and Fig. 1). More students than preceptors agreed that V4 (student = 75%; preceptor = 60%, *p* = 0.012) and V14 (student = 68%; preceptor = 52%, *p* = 0.011) constituted learner mistreatment. Compared to students, more preceptors agreed that V2 (student = 59%; preceptor = 77%, *p* = 0.002), V9 (student = 69%; preceptor = 93%, *p* < 0.001), and V12 (student = 47%; preceptor = 63%, *p* = 0.011) demonstrated mistreatment behaviors.

Table 3 presents the percentages of medical students and preceptors who selected the neutral response for each vignette. The proportion of neutral responses was higher among preceptors for ten of the 17 vignettes (V1, V3, V4, V7, V8, V10, V13, V14, V16, and V17). Compared to preceptors, a greater proportion of students had neutral responses to V9 (student = 15%; preceptor = 5%, *p* < 0.013).

**Table 3:** The proportion of students and preceptors (i.e., Faculty, Residents, Fellows, and Other) who selected “neutral” in each vignette and results from Fisher’s exact tests testing the difference in the proportion of neutral responses between students and preceptors in each vignette (*n* = 271)

Vignette	Student	Preceptor	*p* value
	(*n* = 100)	(*n* = 171)	
**V1: Limited Ob/Gyn participation for male student (%)**			0.755
Not neutral	81 (81)	135 (79)	
Neutral	19 (19)	36 (21)	
**V2: "Don’t worry, Honey." (%)**			0.861
Not neutral	84 (84)	146 (85)	
Neutral	16 (16)	25 (15)	
**V3: Limited OR participation for female student (%)**			1.000
Not neutral	85 (85)	144 (84)	
Neutral	15 (15)	27 (16)	
**V4: "You should know the answer to that." (%)**			0.195
Not neutral	86 (86)	136 (80)	
Neutral	14 (14)	35 (20)	
**V5: Student's preferred pronouns (%)**			1.000
Not neutral	94 (94)	160 (94)	
Neutral	6 (6)	11 (6)	
**V6: EKG interpretation (Control) (%)**			0.186
Not neutral	88 (88)	159 (93)	
Neutral	12 (12)	12 (7)	
**V7: "I bet you were a great athlete." (%)**			0.564
Not neutral	77 (77)	125 (73)	
Neutral	23 (23)	46 (27)	
**V8: Family planning and career choice (%)**			0.332
Not neutral	75 (75)	118 (69)	
Neutral	25 (25)	53 (31)	
**V9: Persisting approach from faculty (%)**			**0.013**
Not neutral	85 (85)	162 (95)	
Neutral	15 (15)	9 (5)	
**V10: Misleading report from resident (%)**			1.000
Not neutral	95 (5)	161 (94)	
Neutral	5 (5)	10 (6)	
**V11: Caregiving tasks (%)**			1.000
Not neutral	84 (84)	143 (84)	
Neutral	16 (16)	28 (16)	
**V12: Limited OR participation due to career choice (%)**			0.534
Not neutral	77 (77)	138 (81)	
Neutral	23 (23)	33 (19)	
**V13: Orientation for selected group of students (%)**			0.408
Not neutral	74 (74)	117 (68)	
Neutral	26 (26)	54 (32)	
**V14: Wrong student name (%)**			0.270
Not neutral	75 (75)	117 (68)	
Neutral	25 (25)	54 (32)	
**V15: Manuscript authorship (%)**			0.064
Not neutral	96 (96)	170 (99)	
Neutral	4 (4)	1 (1)	
**V16: Interjecting feedback-English fluency (%)**			0.063
Not neutral	92 (92)	143 (84)	
Neutral	8 (8)	28 (16)	
**V17: Missed training opportunity (%)**			0.241
Not neutral	80 (80)	125 (73)	
Neutral	20 (20)	46 (27)	

*n*, observations; *Ob/Gyn*, obstetrics and gynecology; *EKG*, electrocardiogram; *OR*, operating room

## Discussion

Students’ and preceptors’ perceptions did not differ significantly for 13 of the 17 vignettes that were employed in this study. Nearly all participating students and preceptors agreed that one vignette describing the denial of a training opportunity based on gender (V11) and one example of being denied a training opportunity based on training stage (V15) constituted learner mistreatment. Most students and preceptors agreed that one case with comments regarding gender groups (V5), and one case of interjecting feedback about language fluency during a student presentation (V16) constituted learner mistreatment. For these cases, shared mistreatment perceptions among preceptors and students could reflect institution-specific training on appropriate behavior in the learning environment. The majority of students and preceptors responded similarly to V6 (EKG interpretation-control and V10 (misleading report from resident), which is consistent with findings observed in prior studies that employed these cases [24, 29].

Discrepancies in students’ and preceptors’ perceptions of mistreatment were observed for five cases. A higher proportion of preceptors agreed that mistreatment behaviors that could be classified as overt, such as persistent requests to meet with a preceptor outside of work settings (V9) and denial of a training opportunity due to career choice (V12), constituted learner mistreatment. More students agreed that being publicly embarrassed (V4) and being addressed with another student’s name (V14) constituted learner mistreatment, suggesting that students may respond differently to cases that may be more subtle, or in scenarios that are employed less often in mistreatment literature [24]. This finding aligns with prior studies that showed disagreement between students and preceptors regarding mistreatment behaviors that are less overt or that may be shaped by a broader range of contextual factors [16, 23, 24].

Unique to this investigation, we paid special attention to vignettes that illustrated AAMC-defined negative behaviors in order to determine whether these influence whether students’ and preceptors’ mistreatment perceptions align. This was supported by our findings, as differing perceptions were observed in cases that did not resemble AAMC-defined mistreatment behaviors. These results may reflect students’ and preceptors’ familiarity or ability to identify AAMC-defined negative behaviors [7, 19, 38]. Future research should elaborate on factors that shape preceptors’ and students’ interpretations of student-faculty interactions, in order to identify which behaviors most affect decisions about whether mistreatment has taken place, as well as what type of conduct needs addressing, and what interventions are most appropriate [38].

With respect to language, more faculty agreed that addressing students with the term “honey” (V12) constituted learner mistreatment. Hildebrand and colleagues’ [39] showed that “terms of endearment” may be perceived differently by gender. In another study, more female academic hospitalists reported being addressed with terms of endearment than their male counterparts, and participants believed this behavior was disrespectful towards their role in medicine [40]. Further examination of how terms of endearment are used and perceived in medical training may lend important insights on acceptability of this type of behavior, with potential considerations for gender, role, institution, and region.

Not surprisingly, a greater proportion of students (75%) than preceptors (60%) agreed that the case describing a student who may feel embarrassed in front of an audience of peers (V4) constituted learner mistreatment. The proportion of students who considered V4 an example of learner mistreatment may reflect students’ potentially overlapping classification of “public embarrassment” and “public humiliation” reported in prior scholarship [41, 42]. As preceptors may employ Socratic teaching tenets to publicly engage learners in question-based dialog, a student may experience embarrassment if they cannot answer correctly [43]. Several studies have linked public humiliation to specific negative behaviors [44–47], yet learners and preceptors may require structured explanations of expectations along with mistreatment definitions to effectively differentiate public embarrassment and public humiliation [48, 49]. Additional efforts that reinforce definitions of public embarrassment and public humiliation, including the role of intent, are warranted. Professional development on appropriate bedside teaching methods, as well as learner orientation to the purpose of various educational tactics, may decrease conflated reports of public embarrassment and public humiliation events [41].

While most faculty (88%) and students (88%) agreed that disregarding a student’s preferred pronouns constitutes learner mistreatment (V5), this type of behavior is rarely featured in studies and training content regarding gender-related mistreatment. As current definitions of gender- or identity-related mistreatment may not explicitly list preferred pronouns, this finding calls for further investigation and potential revision of mistreatment definitions, categories, and resources to incorporate language preferences [3, 50, 51]. In addition, offering students and preceptors training related to acknowledging the language preferences of their colleagues and peers, as well as incorporating policies that guide this type of professional behavior in the learning environment, is warranted [52–54].

Additional analysis of students’ and preceptors’ selection of “neutral” responses generated important findings. Over 25% of students and faculty remained neutral about whether offering an orientation session for a traditionally minoritized group of students (V13) constituted learner mistreatment. More than a quarter of preceptors selected neutral responses for three additional vignettes (V8, V14, and V17). More students than preceptors were selected neutral responses for V9, in which a preceptor repeatedly asks a student to meet socially outside of the clinical setting. While neutral responses may indicate indecisiveness due to absent contextual information [36], further research and education about how and when learners and preceptors make decisions about mistreatment could generate additional factors that contribute to, or impede mistreatment decisions [25, 38]. The concept of “neutrality” or choosing to remain neutral toward a learning environment scenario has not been published extensively in mistreatment literature, and this study’s findings suggest instances in which learners and preceptors may or may not make decisions about whether a learner has been mistreated.

This study’s findings should be interpreted in light of several limitations; as results are based on data from one institution, vignettes were not designed to illustrate all potential forms of mistreatment behaviors, and not all vignettes included behaviors that met existing parameters for negative behaviors [32]. Additionally, social desirability bias may have prompted participants to select answers that do not reflect their true interpretation of the vignettes [55]. Lastly, the survey instructions did not ask respondents to adopt a particular position (e.g., student, preceptor, third-party observer); rather, participants were only instructed to identify whether a behavior in a scenario constituted learner mistreatment. However, the influence of participants’ real or imagined role, as well as influences of past or present training experiences on their responses, is an important area for further exploration.

These findings add a unique contribution to ongoing mistreatment scholarship by exploring perceptions of written vignettes among students and preceptors from a Southern USA MD-granting institution, including whether respondents made decisions about mistreatment at all (i.e., “neutral” response). The survey tool used in this study incorporated adapted cases from prior vignette studies and prior mistreatment investigations, as well as locally developed cases, and the sample was not limited to persons who have experienced or previously reported mistreatment, which may better represent the general population [15, 16, 23, 24, 29, 31].

## Conclusion

The lack of consensus reported in this study is a key finding, as students’ and preceptors’ divergent views on behaviors that constitute learner mistreatment may interfere with identification, decisions to report, and potential intervention, thus perpetuating cycles of unaddressed mistreatment [19, 38]. Students’ and preceptors’ perceptions primarily differed in areas in which existing definitions may be less explicit; prior training regarding professional conduct, as well as individual experiences, may in part explain differing views that were observed [16, 24]. Shared definitions of mistreatment behaviors supply learners, faculty, and administrators with clear concepts and descriptions (e.g., harassment, discrimination) to monitor the learning environment and distinguish behaviors that interrupt the learning process. As learners’ training experiences may still be disturbed by behaviors that may not fit commonly accepted mistreatment definitions, such as unfavorable treatment based on career choice or other characteristics [3, 51, 56, 57], ongoing work is necessary to mitigate gaps and create resources based on a shared understanding of appropriate behavior, including contextual factors, particularly in health professions-specific learning environments.

Highlighted areas of discrepancy reported in this study can inform educational exploration and intervention to increase awareness, refine definitions, enhance shared understanding, and ideally decrease incidence of mistreatment in health professions training environments [22, 58]. Follow-up research is essential for understanding perception, interpretation, and decision-making to improve experiences for all stakeholders in the learning environment.

## Data Availability

Data supporting the findings of this study are available from the corresponding author, ACL, upon reasonable request.

## References

[1] Cook AF, Arora VM, Rasinski KA, Curlin FA, Yoon JD. The prevalence of medical student mistreatment and its association with burnout. Acad Med. 2014;89(5):749–54. 10.1097/ACM.0000000000000204. 24667503 10.1097/ACM.0000000000000204 PMC4401419

[2] Frank E, Carrera JS, Stratton T, Bickel J, Nora LM. Experiences of belittlement and harassment and their correlates among medical students in the United States: longitudinal survey. BMJ. 2006;333(7570):682. 10.1136/bmj.38924.722037.7C. 16956894 10.1136/bmj.38924.722037.7C PMC1584373

[3] Hill KA, Samuels EA, Gross CP, et al. Assessment of the prevalence of medical student mistreatment by sex, race/ethnicity, and sexual orientation. JAMA Intern Med. 2020;180(5):653–65. 10.1001/jamainternmed.2020.0030. 32091540 10.1001/jamainternmed.2020.0030 PMC7042809

[4] Kassebaum DG, Cutler ER. On the culture of student abuse in medical school. Acad Med. 1998;73(11):1149–58. 10.1097/00001888-199811000-00011. 9834696 10.1097/00001888-199811000-00011

[5] Silver HK, Glicken AD. Medical student abuse: incidence, severity, and significance. JAMA. 1990;263(4):527–32. 10.1001/jama.1990.03440040066030. 2294324

[6] Henning MA, Stonyer J, Chen Y, et al. Medical students’ self-perceptions of harassment during clinical placement. Med Sci Educ 2023/10/27 2023. 10.1007/s40670-023-01926-5 10.1007/s40670-023-01926-5 PMC10948714 38510407

[7] AAMC. Medical School Graduation Questionnaire. All School Summary Report. 2023.

[8] Schuchert MK. The relationship between verbal abuse of medical students and their confidence in their clinical abilities. Article Academic Medicine. 1998;73(8):907–9. 10.1097/00001888-199808000-00018. 9736853 10.1097/00001888-199808000-00018

[9] Elnicki DM, Curry RH, Fagan M, et al. Medical students’ perspectives on and responses to abuse during the internal medicine clerkship. Teach Learn Med. 2009;14(2):92–7. 10.1207/S15328015TLM1402_05. 10.1207/S15328015TLM1402_05 12058552

[10] Heru A, Gagne G, Strong D. Medical student mistreatment results in symptoms of posttraumatic stress. Academic Psychiatry. 2009;33(4):302–6. 10.1176/appi.ap.33.4.302. 19690110 10.1176/appi.ap.33.4.302

[11] Oser TK, Haidet P, Lewis PR, Mauger DT, Gingrich DL, Leong SL. Frequency and negative impact of medical student mistreatment based on specialty choice: a longitudinal study. Acad Med. 2014;89(5):755–61. 10.1097/acm.0000000000000207. 24667501 10.1097/ACM.0000000000000207

[12] Richman JA, Flaherty JA, Rospenda KM, Christensen ML. Mental health consequences and correlates of reported medical student abuse. JAMA. 1992;267(5):692–4. 10.1001/jama.1992.03480050096032. 1731137

[13] Crombie KE, Crombie KD, Salie M, Seedat S. Medical students’ experiences of mistreatment by clinicians and academics at a South African university. Teach Learn Med 2023;1–10. 10.1080/10401334.2023.2167207 10.1080/10401334.2023.2167207 36647677

[14] Barrett J, Scott KM. Acknowledging medical students’ reports of intimidation and humiliation by their teachers in hospitals. J Paediatr Child Health. 2018;54(1):69–73. 10.1111/jpc.13656. 28767175 10.1111/jpc.13656

[15] Fleit HB, Lu W-H, Olvet DM, Chandran L. Medical students’ perception of behaviors in the clinical learning environment evolve with increasing clinical exposure as measured with situational video vignettes. Med Teach. 2020;42(7):822–7. 10.1080/0142159X.2020.1759790. 32401093 10.1080/0142159X.2020.1759790

[16] Kulaylat AN, Qin D, Sun SX, et al. Perceptions of mistreatment among trainees vary at different stages of clinical training. BMC Med Educ. 2017;17(1):14. 10.1186/s12909-016-0853-4. 28088241 10.1186/s12909-016-0853-4 PMC5237524

[17] Williams-Karnesky RL, Russell JC, Wang M-L. More than aligning perception: impact of an educational intervention on medical student mistreatment reporting. J Am Coll Surg. 2020;231(1):112-121e2. 10.1016/j.jamcollsurg.2020.03.029. 32283271 10.1016/j.jamcollsurg.2020.03.029

[18] AAMC. Medical School Graduate Questionnaire: 2022 All Schools Summary Report. 2022. https://www.aamc.org/media/55736/download

[19] Bell A, Cavanagh A, Connelly CE, Walsh A, Vanstone M. Why do few medical students report their experiences of mistreatment to administration? Med Educ. 2021;55(4):462–70. 10.1111/medu.14395. 33063354 10.1111/medu.14395

[20] Chung MP, Thang CK, Vermillion M, Fried JM, Uijtdehaage S. Exploring medical students’ barriers to reporting mistreatment during clerkships: a qualitative study. Med Educ Online. 2018;23(1):1478170. 10.1080/10872981.2018.1478170. 29848223 10.1080/10872981.2018.1478170 PMC5990956

[21] Fried JM, Vermillion M, Parker NH, Uijtdehaage S. Eradicating medical student mistreatment: a longitudinal study of one institution’s efforts. Acad Med. 2012;87(9):1191–8. 10.1097/ACM.0b013e3182625408. 22836847 10.1097/ACM.0b013e3182625408 PMC4399975

[22] Kulaylat AN, Qin D, Sun SX, et al. Aligning perceptions of mistreatment among incoming medical trainees. J Surg Res. 2017;208:151–7. 10.1016/j.jss.2016.09.016. 27993202 10.1016/j.jss.2016.09.016

[23] Ogden PE, Wu EH, Elnicki MD, et al. Do attending physicians, nurses, residents, and medical students agree on what constitutes medical student abuse? Acad Med. Oct2005;80(10 Suppl):S80–3. 10.1097/00001888-200510001-00022. 16199465 10.1097/00001888-200510001-00022

[24] Peckston DC, Urwin R, McMullan R, Westbrook J. Student and clinician perceptions of medical student mistreatment: a cross-sectional vignette survey. BMJ Open. 2022;12(9):e061253. 10.1136/bmjopen-2022-061253. 10.1136/bmjopen-2022-061253 PMC9476143 36104130

[25] Fleit HB, Lu W-H, Olvet DM, Chandran L. Case studies for recognizing appropriate and inappropriate behaviors in the clinical learning environment. MedEdPORTAL. 2017;13:10638. 10.15766/mep_2374-8265.10638. 30800839 10.15766/mep_2374-8265.10638 PMC6338161

[26] Feldman NL, Lewis JL, Patel CK, et al. The other side of medical student mistreatment: teaching cultural competency across the generational divide. MedEdPORTAL. 2019;15:10847. 10.15766/mep_2374-8265.10847. 31921993 10.15766/mep_2374-8265.10847 PMC6946582

[27] Holmström SW, Klocksieben FA, Forrester LD, Zreibe D, O’Brien KE. Medical student mistreatment—an obstetrics and gynecology perspective: a pilot study. Med Sci Educ. 2019;29(3):787–94. 10.1007/s40670-019-00740-2. 34457543 10.1007/s40670-019-00740-2 PMC8368386

[28] AAMC. Medical School Graduation Questionnaire. All School Summary Report. 2022.

[29] Ellis S, Purkiss J, Abdoler E, et al. Variability in student perceptions of mistreatment. Clin Teach. 2019;16(2):142–6. 10.1111/tct.12790. 29786958 10.1111/tct.12790

[30] Medicine USo. Understanding and reporting mistreatment. https://hsc.unm.edu/medicine/education/leo/reporting/

[31] Kristoffersson E, Andersson J, Bengs C, Hamberg K. Experiences of the gender climate in clinical training – a focus group study among Swedish medical students. BMC Med Educ. 2016;16(1):283. 10.1186/s12909-016-0803-1. 27784300 10.1186/s12909-016-0803-1 PMC5082355

[32] AAMC. Medical School Graduate Questionnaire: 2023 All Schools Summary Report. 2023.

[33] Qualtrics. Version 2023. 2020. https://www.qualtrics.com

[34] Harpe SE. How to analyze Likert and other rating scale data. Curr Pharm Teach Learn. 2015;7(6):836–50. 10.1016/j.cptl.2015.08.001.

[35] Pagano M, Halvorsen KT. An algorithm for finding the exact significance levels of r × c contingency tables. J Am Stat Assoc. 1981;76(376):931–4. 10.2307/2287590.

[36] Hughes R, Huby M. The construction and interpretation of vignettes in social research. Soc Work Soc Sci Rev. 2012;11(1):36–51. 10.1921/swssr.v11i1.428.

[37] StataCorp. Stata Statistical Software: Release 18. College Station, TX: StataCorp LLC; 2023.

[38] Vanstone M, Cavanagh A, Molinaro M, et al. How medical learners and educators decide what counts as mistreatment: a qualitative study. Med Educ 2023;57(10). 10.1111/medu.15065 10.1111/medu.15065 36815430

[39] Hildebrand LK, Monteith MJ, Carter ER, Burns MD. Honey, sweetie, dear: terms of endearment communicate, reflect, and reinforce sexism toward adult women. Sex Roles. 2022;87(3):185–210. 10.1007/s11199-022-01311-3.

[40] Bhandari S, Jha P, Cooper C, Slawski B. Gender-based discrimination and sexual harassment among academic internal medicine hospitalists. J Hosp Med. 2021;16(2):84–9. 10.12788/jhm.3533. 33496657 10.12788/jhm.3533

[41] Markman JD, Soeprono TM, Combs HL, Cosgrove EM. Medical student mistreatment: understanding “public humiliation.” Med Educ Online. 2019;24(1):1615367–1615367. 10.1080/10872981.2019.1615367. 31066349 10.1080/10872981.2019.1615367 PMC6507954

[42] Jarukasemkit S, Kaewkamjornchai P, Tam KM. System dynamics modeling to understand mental model of public humiliation in medical education. Med Teach. 2022;44(8):872–7. 10.1080/0142159X.2022.2041587. 35271406 10.1080/0142159X.2022.2041587

[43] Kost A, Chen FM. Socrates was not a pimp: changing the paradigm of questioning in medical education. Acad Med. 2015;90(1):20–4. 10.1097/ACM.0000000000000446 25099239

[44] Heidi L, Clive S. The hidden curriculum in undergraduate medical education: qualitative study of medical students’ perceptions of teaching. BMJ. 2004;329(7469):770. 10.1136/bmj.329.7469.770. 15459051 10.1136/bmj.329.7469.770 PMC520997

[45] Phillips SP, Clarke M. More than an education: the hidden curriculum, professional attitudes and career choice. Med Educ. 2012;46(9):887–93. 10.1111/j.1365-2923.2012.04316.x. 22891909 10.1111/j.1365-2923.2012.04316.x

[46] Rees CE, Monrouxe LV. “A morning since eight of just pure grill”: a multischool qualitative study of student abuse. Acad Med. 2011;86(11):124–33. 10.1097/ACM.0b013e3182303c4c 21952053

[47] Anderson JIM. Can “pimping” kill? The potential effect of disrespectful behavior on patient safety. JAAPA. 2013;26(4):53–6. 10.1097/01720610-201304000-00014 23610843

[48] Stoddard HA, O’Dell DV. Would Socrates have actually used the “Socratic method” for clinical teaching? J Gen Intern Med. 2016;31(9):1092–6. 10.1007/s11606-016-3722-2. 27130623 10.1007/s11606-016-3722-2 PMC4978680

[49] Gan R, Snell L. When the learning environment is suboptimal: exploring medical students’ perceptions of “mistreatment”. Acad Med. 2014;89(4):608–17. 10.1097/ACM.0000000000000172 PMC4885564 24556767

[50] Filimonov AK, Gates AR, Allos AN, Billings HJ, Goldina A, Wisco JJ. A call to action for improving LGBTQIA2S+ inclusive policies and practices in educating science and medical professionals. Med Sci Educ. 2023;33(3):767–72. 10.1007/s40670-023-01797-w. 10.1007/s40670-023-01797-w PMC10368593 37501802

[51] Samuels EA, Boatright DH, Wong AH, et al. Association between sexual orientation, mistreatment, and burnout among US medical students. JAMA Netw Open. 2021;4(2):e2036136–e2036136. 10.1001/jamanetworkopen.2020.36136. 33528552 10.1001/jamanetworkopen.2020.36136 PMC7856540

[52] Gacita A, Gargus E, Uchida T, et al. Introduction to safe space training: interactive module for promoting a safe space learning environment for LGBT medical students. MedEdPORTAL. 2017;13:10597. 10.15766/mep_2374-8265.10597. 30800799 10.15766/mep_2374-8265.10597 PMC6338189

[53] Freeman NW, Keuroghlian AS. Supporting transgender and gender diverse medical students. The Lancet. 2022;400(10355):804–5. 10.1016/S0140-6736(22)01698-1. 10.1016/S0140-6736(22)01698-1 36088942

[54] Baecher-Lind L, Sutton JM, Bhargava R, et al. Strategies to create a more gender identity inclusive learning environment in preclinical and clinical medical education. Acad Med. 2023;98(12):1351–5 10.1097/ACM.0000000000005334 37478137

[55] Krumpal I. Determinants of social desirability bias in sensitive surveys: a literature review. Qual Quant. 2013;47(4):2025–47. 10.1007/s11135-011-9640-9.

[56] Woolley DC, Paolo AM, Bonaminio GA, Moser SE. Student treatment on clerkships based on their specialty interests. Teach Learn Med. 2006;18(3):237–43. 10.1207/s15328015tlm1803_9. 16776612 10.1207/s15328015tlm1803_9

[57] Woolley DC, Moser SE, Davis NL, Bonaminio GA, Paolo AM. Treatment of medical students during clerkships based on their stated career interests. Teach Learn Med. 2003;15(3):156–62. 10.1207/S15328015TLM1503_02. 12855385 10.1207/S15328015TLM1503_02

[58] Heru AM. Using role playing to increase residents' awareness of medical student mistreatment. Acad Med. 2003;78(1):35–8. 10.1097/00001888-200301000-00008 12525407

